# Elastic Laminal Invasion in Colon Cancer: Diagnostic Utility and Histological Features

**DOI:** 10.3389/fonc.2012.00179

**Published:** 2012-12-11

**Authors:** Motohiro Kojima, Mitsuru Yokota, Norio Saito, Shogo Nomura, Atsushi Ochiai

**Affiliations:** ^1^Pathology Field, Research Center for Innovative Oncology, National Cancer Center Hospital EastKashiwa, Chiba, Japan; ^2^Division of Pelvic Surgery, National Cancer Center Hospital EastKashiwa, Chiba, Japan; ^3^Clinical Trial Section, Research Center for Innovative Oncology, National Cancer Center Hospital EastKashiwa, Chiba, Japan

**Keywords:** colon cancer, pathology, diagnosis, elastic lamina, tumor spread

## Abstract

Primary tumors of the colorectal cancers are assessed pathologically based on the tumor spread into the bowel wall. The assessment of serosal involvement, which may be relevant to pT4, can be challenging for pathologists, making the consistency of diagnoses questionable. As solutions to this problem, the following two strategies could be adopted. One would be to use special staining or immunohistochemical staining techniques for diagnostic assistance. The other would be to construct recommendations for the assessment of tumor spreading and to obtain a world-wide consensus on the criteria used to assess tumor spreading. Using elastic staining, we previously reported that peritoneal elastic laminal invasion (ELI) could be objectively determined and would likely contribute to a simplified and more objective stratification of deep tumor invasion around the peritoneal surface. We also noted the importance of sampling, staining, and histo-anatomical knowledge in the application of elastic staining during routine pathological diagnosis. Here we review the history of primary tumor stratification leading to the present TNM classification and report on the current status of pathological assessments made at our hospital to summarize what has been established and what is further required for the pathological diagnosis of tumor spreading in patients with colorectal cancer.

## Introduction

Since the first categorization efforts reported by Lockhart-Mummery (1926–1927), primary colorectal cancers have been consistently stratified based on the extent of their spreading into the bowel wall (Dukes, 1932; Jass et al., 1987; Newland et al., 1995). Deep tumor invasion around the peritoneal surface has also been reported as a prognostic factor (invasion through all the layers, peritoneal involvement, or direct spreading involving a free serosal surface; Astler and Coller, 1954; Shepherd et al., 1997). These reports were base for the 7th TNM classification (Sobin et al., 2009). Reviewing these reports should help to renew our understanding of what has been established and what is further required for the pathological diagnosis of tumor spreading. Using elastica staining, we previously showed that peritoneal elastic laminal invasion (ELI) could be objectively determined and would likely contribute to a simplified and more objective stratification of deep tumor invasion around the peritoneal surface (Kojima et al., 2010). We also note the importance of sampling, staining, and histo-anatomical knowledge to apply elastica staining to routine pathological diagnosis. In this review, we will reflect on the brilliant achievements in the assessment of tumor spreading in colorectal cancer. We will also report on the current status of pathological assessments made at our hospital and will expose associated problems that will require solutions in the future. In addition to the diagnostic criteria, we also feel that a comprehensive minimum consensus is required for pathology protocols, including sampling and staining protocols in the future. Next, in addition to their prognostic relevance, areas with ELI exhibit marked fibrosis and tumor budding. These findings suggest that ELI areas may actively induce metastasis and such observations may lead to future biological investigations. On the other hand, ELI can only be a surrogate marker of deep tumor invasion. Therefore, we reviewed our records and showed that ELI was a superior prognostic marker compared with the depth of tumor invasion, suggesting that the ELI tumor area may play an active role in the metastasis of colorectal cancer. Finally, we summarize several biological topics that may be relevant to our pathological findings.

## History of the Assessment of Tumor Spreading in Colorectal Cancer

Many early reports of the classification of tumor spreading focused mainly on rectal cancer. In the first report by Lochart-Mummery, tumor spreading and metastasis was classified as follows: (A) favorable cases, tumor did not invade the muscularis; no nodes involved; (B) medium cases, tumor invaded muscular coat; no extensive involvement of nodes; and (C) very bad cases, tumor large, and fixed; or extensive involvement of nodes. In the classical Duke’s classification, tumor spreading was classified according to the presence of extra-rectal tissue. And in the modified Duke’s classification published in 1958, tumor spreading was classified as (1) confined to the bowel wall, (2) commencing to invade the extra-rectal tissues, (3) well established in the mesentery, or (4) deeply invasive, possibly into neighboring organs. Also, in the modified Duke’s classification reported by Kirklin et al. (1949) divided as follows: type A, lesion limited to the mucosa; Type B1, lesion extended into the muscularis propria, but not penetrating it; Type B2, lesion penetrated through the muscularis propria. They completed the construction of an outline for the current pT1-3 stages in the current TNM classification. They also found that the extent of local spreading was associated with a poor 5 year survival rate, and their report was followed by studies measuring the depth of local spreading in rectal cancer (Cawthorn et al., 1990; Shirouzu et al., 2011). As reported by Kirklin et al. we now know that most of these classifications can also be used for the assessment of colon cancer. On the other hand, some histo-anatomical differences exists, the largest is the existence of the peritoneal coat, leading to differences in the pathological criteria for pT4 colon cancer, compared with those used for rectal cancer. Free mesothelial surface involvement or local peritoneal involvement (lPI), which relevant to present pT4 was reported by Newland et al. (1993) and Shepherd et al., 1995; Ludeman and Shepherd, 2005). They challenged to sub-stage tumor spreading beyond the bowel wall using the peritoneal surface. Using the modified Australian ClinicoPathological Staging System (ACPS), Newland et al. showed that tumor spreading involving a free mesothelial surface was a prognostic factor. Shepherd et al. showed that LPI Group 3 and 4 were predictors of a poor prognosis in patients with colorectal cancer. Apart from the details of these definitions, they found colorectal cancer spreading just around or over the outer surface of the bowel wall was associated with a poor prognosis, and many data support their criteria (we termed “tumor involvement of free mesothelial surface,” “pT4,” and “LPI Group 3 and 4” as serosal involvement in following context). Using a fully standardized pathology, other strategies for the treatment of colorectal cancer may be developed (Wolpin and Mayer, 2008). For example, adjuvant chemotherapy may provide a benefit to colonic cancer patients with serosal involvement who do not have lymph node metastasis (stage IIB; Morris et al., 2007).

## Current Status of Macroscopic and Histological Examination to Assess Serosal Involvement and Exposure of Diagnostic Problems

**Figure 1:**
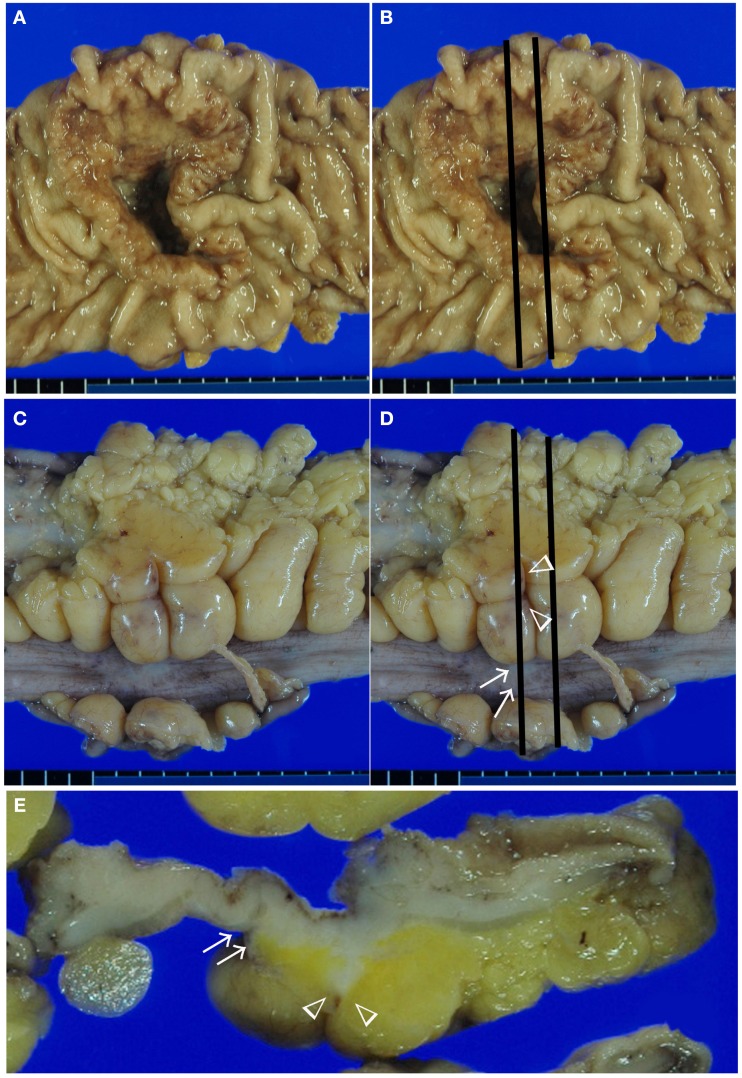
**Macroscopic assessment of serosal invasion in colonic cancer**. Macroscopic features from luminal side **(A,B)**, serosal side **(C,D)**, and cut surface of the lesion **(E)**. In this case, we identified both a serosal indentation (arrow) and the widening and brownish change of the crevice (arrow head) in **(D)**. The first two slices through these lesions are shown in **(B,D)**, and the cut surface is shown in **(E)**. On the cut surface, the indentation of the serosal surface (arrow) and a streak sign (arrow head) were identified. These areas were sampled for histological examination in Figure 2.

Detailed macroscopic observation and sampling are essential steps in making an accurate and objective diagnosis. Macroscopic features from luminal and serosal side were shown in Figures 1A,C, respectively. The slices we made are shown in black line in Figures 1B,D. In our department, surgically resected specimens are extended using a pin and cork board. After 24 h of fixation in 10% buffered formalin, macroscopic observations of the luminal, and serosal sides are performed, and the deepest area of the ulcer floor and indentations of the serosal surface are identified. In the tenial area of the colonic wall, serosal indentation is easy to identify (Figure 1D, arrow). On the other hand, serosal involvement on the adipose rich mesenteric side is often difficult to identify (Figure 1D, arrow head). In the normal state, fatty appendices of the colon form peritoneum-lined crevice. Serosal involvement, especially free-floating tumor cells are often found in this area. Macroscopically, the existence of serosal involvement in this area can be speculated based on widening or brownish change in the crevice near the deepest area of ulcer floor (Figure 1D, arrow head). Therefore, we make the first cross-sectioning of the tumor in a manner that places the cut through these above-mentioned areas (Figures 1B,D). A parallel slice is then made after the first slice. Indentations of the serosal surface should be identified on the cut surface and sampled extensively (Figure 1E, arrow and arrow head). In addition, case with serosal involvement frequently shows macroscopic stricture (Miyamoto et al., 2001). Serosal involvement can also be observed as a macroscopic streak sign on the cut surface, providing a useful clue for identification (Inomata et al., 1998; Figure 1E, arrow head).

Histologically, many pathologists report tumor spreading according to the definition in TNM classification. However, a recent study has questioned the reproducibility of serosal involvement. Similarly, the diagnosis of pT3 or pT4 is often difficult. This situation has been well described by Stewart et al. (2007b, 2011). They pointed out the histo-anatomical and histopathological characteristics of peritoneal tissue. Histo-anatomically, peritoneal tissue consists of the mesothelium and submesothelial layer, which coats the colonic wall and the surrounding pericolic adipose tissue (Mills, 2007). Therefore, especially around the adipose tissue, the peritoneal surface is not smooth, but instead exhibits peritoneal clefts or peritoneal reflection where serosal involvement is frequently seen (Figure 1C).

**Figure 2:**
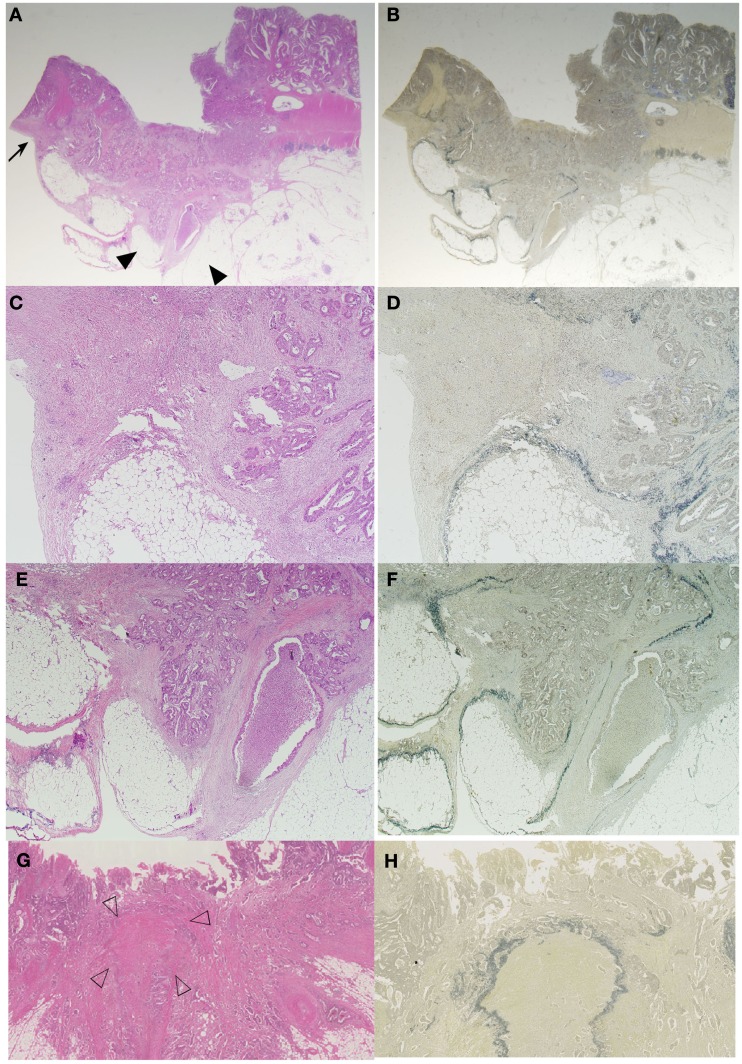
**Histological features of serosal invasion in the case shown in Figure 1**. The arrows and arrow heads in the macroscopic picture shown in Figure 1E are identical to the arrows and arrow heads in the histologic picture shown in **(A,B)**. Elastica staining picture from the serial section was obtained, and H.E stained **(A,C,E,G)** is same area with elastic stained **(B,D,F,H)**, respectively. **(A–F)** Histological features showing peritoneal surface elevation toward the tumor (arrow) and a streak sign (arrow head) in a colorectal cancer specimen. The tumor had deeply invaded the region near the serosal surface, and prominent fibroinflammation was visible in the area, identical to the macroscopic findings **(C,E)**. ELI is also seen in the area with macroscopic findings **(D,F)**. In some cases, prominent fibrosis with a non-cellular matrix component forms a fibrotic focus **(G)**. Using elastica staining we detect fibrotic focus just beneath the peritoneal elastic lamina **(H)**.

**Figure 3:**
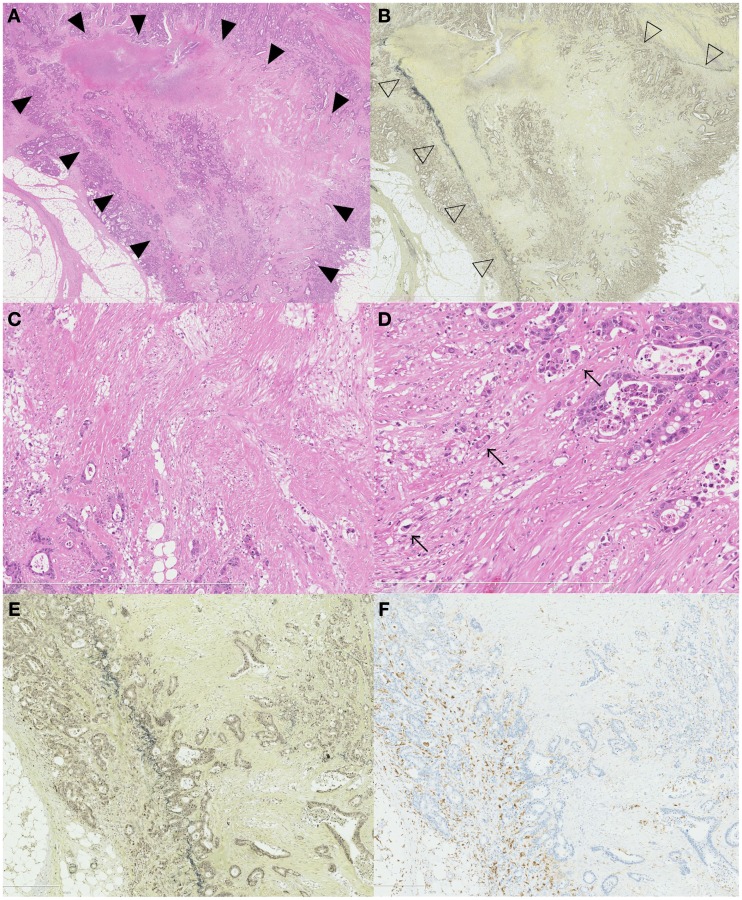
**Association of ELI, fibrotic focus, and macrophage**. A scar like radiating fibrosclerotic core of a fibrotic focus [**(A)**, black arrow head] is found just beneath the peritoneal elastic lamina [**(B)**, white arrow head]. Using high-power magnification, the fibroblasts arranged in a storiform pattern **(C,D)** The distribution of CD204-positive macrophages and an association with the ELI and fibrotic focus is seen in **(E,F)**. CD204-positive macrophages are distributed along the periphery of the fibrotic focus **(E)** and along the peritoneal elastic lamina **(F)**.

Difference in the level of H.E slides and the difference of the elastic staining used can influence on the diagnosis of both serosal invasion and ELI. In our hospital, histological evaluation is performed by two levels of H.E staining and one routine Elastica staining using Maeda Resorcin-Fuchshin Solution as described previously (Kojima et al., 2011). Histological slides of the same tumor section with Figure 1E was shown in Figures 2A–F. Arrow and arrow head in Figure 1E are concordant with that in Figures 2A,B. High power magnification of arrow is corresponded to Figures 2C,D. And that of arrow head is corresponded to Figures 2E,F. We want to stress that histological deepest area of tumor invasion is concordant with macroscopic indentation. Furthermore, peritoneal elastic lamina is retracting toward the tumor (Figures 2B,D,F). Histopathologically, although the normal submesothelial layer contains few cells, this tissue frequently shows fibro inflammatory changes when tumor cells invade areas near this tissue (Figure 2C). In colorectal cancer, fibroinflammation is often seen near the invasive front, but this phenomenon is much more prominent in the tumor area with ELI (Figure 2C). When the fibrosis is prominent, a peritoneal surface elevation toward the tumor is often seen (Figure 2A, arrow). In some cases, prominent fibrosis with a non-cellular matrix component forms a fibrotic focus (Nishimura et al., 1998; Figure 2G). This characteristic morphological features also often found in the tumor area with ELI (Figure 2H). Using low-power observations, a scar-like radiating fibrosclerotic core is observed (Van den Eynden et al., 2007; Figures 3A,B). Using high power magnification, the fibroblasts arranged in a storiform pattern (Figures 3C,D). Marked collagenization is also found. The tumor cell clusters become sparser within area of fibrosis, and budding foci are often seen within this area (Figure 3D). Inflammatory cells, including macrophages, are often seen around these lesions. CD68-positive or CD204-positive macrophages are found predominantly around the peritoneal elastic lamina and fibrotic focus (Figures 3E,F). Such a variety of histopathological alterations makes it difficult to determine serosal involvement by H.E stain alone. Accordingly, even with optimized sampling, the frequency of serosal invasion varies in reports, and the diagnostic concordance is relatively low (Compton, 2003). Therefore, consistent data regarding tumor spreading as assessed at different hospitals is impossible, and this fact hinders the design of multicenter therapeutic trials for high risk stage II colon cancer patients. To overcome this situation, two approaches can be considered. One approach is to establish more detailed diagnostic criteria. Recommendations including assessment and sample preparation protocols may also be useful. In fact, extensive sampling using detailed macroscopic observations definitely improves the accuracy of diagnoses of serosal involvement (Ludeman and Shepherd, 2005). The other approach is to use special staining or immunohistochemical staining techniques to provide diagnostic assistance.

## Ways to Construct Standardized Assessments of Serosal Involvement to Overcome Current Diagnostic Problems

### Establishment of standardized criteria

Pathologists must regularly make difficult choices to diagnose serosal invasion. Diagnostic recommendations or criteria may lessen such difficulties and may even improve the concordance of pathological diagnoses. Any group may decide to establish criteria (informal approach). On the other hand, a criteria established based on more structural surveys would be acceptable for many pathologists, and are increasingly being used to develop clinical guidelines. Klimstra et al. (2010) used this structural survey of “Delphi method” to establish a consensus regarding the reporting of neuroendocrine tumors. Such a method may also contribute to establishing consensus-based criteria for pathological serosal invasion.

### Diagnostic assistance of assessing serosal invasion using elastica staining: Elastic laminal invasion of colon cancer

Special staining and immunohistochemical staining techniques may also be useful for making objective diagnoses of serosal involvement. The peritoneal elastic lamina lies just beneath the subserosal layer. This structure can be visualized using elastica stainings or elastin immunostaining. Although tumor invasion beyond the peritoneal elastic lamina (also known as ELI) is not equal to serosal involvement, based on the position, the presence of deep invasion near the peritoneal surface can be estimated. Recently, the diagnostic utility of ELI as a prognostic marker has been reported. Shinto et al. (2004) firstly reported the utility of ELI for determining high risk patients with pT3 colorectal cancers. They found that pT3 colorectal cancers with ELI (which they termed “pT3 deep”) had similar clinical outcomes to patients with pT4 tumors. Their data was confirmed in our series. On the other hand, the peritoneal elastic lamina is anatomically, the deepest structure from muscular layer. Therefore ELI can only be regarded as a surrogate marker for deep tumor invasion. In this review, we analyzed our previously reported record of 304 patients with curatively resected Stage II or III colonic cancers (Kojima et al., 2010). Using a concordance probability, we evaluated the discriminatory power of ELI for over-all survival and compared that of the depth of tumor invasion. The depth of tumor invasion was measured from the outer border of the muscular layer (Shirouzu et al., 2011). As shown in Table 1, although the concordance probability of ELI was slightly lower than that for the lymph node metastasis, it was much larger than that for the depth of invasion. Therefore, we showed that ELI itself has at least diagnostic utility with more strong predictive power of over-all survival than the depth of tumor invasion. Rather, based on the histological features mentioned above, we estimated that the ELI microenvironment may be capable of promoting tumor progression. Elastin immunostaining was reported not to be more sensitive than elastica staining (Stewart et al., 2007a). Elastica staining is relatively inexpensive and stable method. The detailed examination of vascular invasion is also possible. Therefore this special staining technique can be used as a routine staining method (Abdulkader et al., 2006). Similar to lung cancer, cases with ELI can be classified as different pT entities (Puppa et al., 2011). On the other hand, the peritoneal elastic lamina does not completely cover the colonic wall. The thickness of the elastic lamina also differs depending on the anatomical site (Knudsen, 1991). This fact prompted us, in our previous study, to identify the delicate elastic lamina clearly by following it from one area to another using multiple elastica stainings. And this method made us possible to follow delicate elastic lamina. However, our method may not be practical. And peritoneal elastic lamina may not be detected in more cases in the routine practice (Canney et al., 2012). Furthermore, the staining method used for elastica staining and the number of sections that are stained have not been standardized. We are not yet sure how many slides with elastica staining are needed for a consistent ELI diagnosis. Standard recommendations for the assessment of ELI and serosal invasion are needed for consistent pathological classification.

**Table 1:** **Estimated concordance probability**.

Variables	Concordance probability	SE
Elastic laminal invasion	0.6038	0.0272
Depth of tumor invasion	0.5510	0.0274
Lymph node metastasis	0.6117	0.0276

## Future Perspectives and Hypotheses

### Histological feature of ELI may lead to biological concepts involving the cancer microenvironment

Pathologists can make hypothesis based on histological findings. The presently reported histological features of the ELI area of the tumor may stimulate the imagination of pathologists, possibly leading to medical innovations. Based on our histological findings of fibroinflammation and tumor budding, new hypotheses are likely to be formulated.

### Fibroinflammation and the microenvironment of the ELI tumor area

In addition to the histological features of fibroinflammation in the ELI area, we showed that ELI is strongly associated with distant metastasis, statistically. We have speculated that cancer cells perforate the visceral peritoneum, inducing peritoneal dissemination. However, based on our findings, we speculated that the subserosal microenvironment may provide a special means of actively inducing tumor metastasis. We have found a few previous reports that may available for proving our hypothesis. First, colonic subserosal or other subperitoneal fibroblasts have been cultured *in vitro*. These fibroblasts reportedly produce MCP-1 and VEGF in response to some biological stimuli, such as TGF-beta, IL-1beta, and TNF-alpha, or in response to physiological stimulation such as hypoxia (Witowski et al., 2001; Hirahara et al., 2004; Osada et al., 2009). We speculated that these features of subserosal fibroblast may be associated with the promotion of tumor metastasis in patients with colon cancer. Enhanced reactivity in response to stimuli may be associated with macroscopic indentation or histological fibrosis, and subsequently cancer metastasis. We would like to stress that despite the above-mentioned pathological and biological data, study on the interaction between cancer cells and peritoneal fibroblasts are very rare.

### Biological topics including the tumor microenvironment, cancer stem cells, and EMT, that may be relevant to our pathological findings

We often see tumor budding foci in the ELI area. The microenvironment of ELI can be enriched by chemokines, cytokines, or growth factors, which may induce morphological alterations of the tumor cells (Klampfer, 2011). Budding cells have been reported to share a common phenotype with cancer stem cells or the epithelial mesenchymal transition (Brabletz et al., 2005; Kalluri and Weinberg, 2009). Both concepts are largely biological and we are not sure whether these biological concepts can be accurately compared with pathological morphological concepts, even using the expressions of cancer stem cells or EMT markers (Kojima et al., 2008). However, we now know that budding cells are enriched in the ELI. Therefore, by investigating reciprocal interactions between cancer cells and subserosal fibroblasts, we may be able to estimate the ELI microenvironment and the relevance between tumor budding and the EMT or cancer stem cells.

## Concluding Remarks

We have reflected on the pathological history, examined the current status and problems, and provided future perspectives based on presently available data. We wish to mention that our current clinicopathological works have been supported by the brilliant work of many of our senior pathologists. We believe that an accurate review and recognition of this history will lead to further pathological and biological works for cancer patients.

## Conflict of Interest Statement

The authors declare that the research was conducted in the absence of any commercial or financial relationships that could be construed as a potential conflict of interest.
